# ATH434 Rescues Pre-motor Hyposmia in a Mouse Model of Parkinsonism

**DOI:** 10.1007/s13311-022-01300-0

**Published:** 2022-09-29

**Authors:** Leah C. Beauchamp, Xiang M. Liu, Laura J. Vella, Paul A. Adlard, Ashley I. Bush, David I. Finkelstein, Kevin J. Barnham

**Affiliations:** 1 The Florey Institute of Neuroscience and Mental Health, The University of Melbourne, 30 Royal Parade, Parkville, VIC 3052 Australia; 2 Melbourne Dementia Research Centre, 30 Royal Parade, Parkville, VIC 3052 Australia; 3 Department of Surgery, The Royal Melbourne Hospital, The University of Melbourne, 300 Grattan Street, Parkville, VIC 3050 Australia

**Keywords:** Hyposmia, Parkinson’s disease, Iron, Copper, Prodromal, Tau

## Abstract

Hyposmia is a prevalent prodromal feature of Parkinson’s disease (PD), though the neuropathology that underlies this symptom is poorly understood. Unlike the substantia nigra, the status of metal homeostasis in the olfactory bulbs has not been characterized in PD. Given the increasing interest in metal modulation as a therapeutic avenue in PD, we sought to investigate bulbar metals and the effect of AT434 (formerly PBT434) an orally bioavailable, small molecule modulator of metal homeostasis on hyposmia in a mouse model of parkinsonism (the tau knockout (tau^−/−^) mouse). 5.5 (pre-hyposmia) and 13.5-month-old (pre-motor) mice were dosed with ATH434 (30 mg/kg/day, oral gavage) for 6 weeks. Animals then underwent behavioral analysis for olfactory and motor phenotypes. The olfactory bulbs and the substantia nigra were then collected and analyzed for metal content, synaptic markers, and dopaminergic cell number. ATH434 was able to prevent the development of hyposmia in young tau^−/−^ mice, which coincided with a reduction in bulbar iron and copper levels, an increase in synaptophysin, and a reduction in soluble α-synuclein. ATH434 was able to prevent the development of motor impairment in aged tau^−/−^ mice, which coincided with a reduction in iron levels and reduced neurodegeneration in the substantia nigra. These data implicate metal dyshomeostasis in parkinsonian olfactory deficits, and champion a potential clinical benefit of ATH434 in both prodromal and clinical stages of PD.

**Supplementary Information:**

The online version contains supplementary material available at 10.1007/s13311-022-01300-0.

## Introduction

Idiopathic Parkinson’s disease (PD) is the fastest growing neurological disorder in the world [1] and is characterized by a progressive loss of dopaminergic neurons in the *substantia nigra pars compacta* (SNpc), and the presence of α-synuclein (α-syn) rich inclusions in the remaining neurons (Lewy pathology). The precise mechanisms that lead to the formation of Lewy pathology or nigral neurodegeneration are not known; however, there is increasing evidence that oxidative stress is a key pathological change that leads to impairment of susceptible neurons [2]. There is a selective vulnerability of the SNpc in PD, which is hypothesized to be the result of co-localization of the monoamine dopamine with redox-active metal ions, such as iron (Fe) and copper (Cu) [3, 4]. The reaction of dopamine and metal ions form peroxides and other reactive oxygen species (ROS) [5], which unchecked can lead to catastrophic oxidative damage. These observations have been made in post-mortem human PD SNpc [6, 7] and recapitulated in multiple animal models of disease [8]. These nigral-specific changes are associated with the progressive movement dysfunction associated with PD. However, it is increasingly recognized that there is an extensive prodromal period of the disease in which many non-motor symptoms (NMS) arise, including gastrointestinal (GI) dysfunction, anxiety, sleep disorders, and hyposmia [9]. These NMS are an underrecognized burden that impact on the quality of life for those living with PD and are not well controlled by current therapies [10].

Hyposmia is the most common NMS, affecting as many as 90% of people with PD [11], and often precedes the onset of motor symptoms by decades [12]. The cause of hyposmia in PD is unknown and many observations to date have been made in post-mortem tissue, which is reflective of end-stage disease and may not faithfully reflect the early pathological changes. To overcome this, we have previously demonstrated that the tau knockout (tau^−/−^) mice develop pre-motor hyposmia and we have proposed them as a model of prodromal PD research [13]. These mice develop an autophagy deficit and accumulate α-syn in the olfactory bulb (OB), synonymous with midbrain pathology that develops later in the disease course. Previous work has demonstrated that tau^−/−^ mice have an age-dependent motor phenotype that is accompanied by iron accumulation and neurodegeneration in the SNpc [14, 15]. Increased nigral iron is a widely reported feature of PD [16–18], and given the pathological parallels found between the olfactory system and the midbrain, there is a need to examine the status of metal homeostasis in the OB.

To date, there has only been one published examination of the metal content in the human OB of PD, which demonstrated an increase in the levels of Fe [19]. Interestingly, it is known that metal workers and individuals with occupational exposure to metals have significantly impaired olfaction compared to the general population [20, 21]. Whilst these data are not specific to PD, they demonstrate the potential of metals to disrupt normal olfactory processing which is of great interest in diseases with known metal dyshomeostasis, such as PD.

Metal modulation is a viable therapeutic target in neurodegenerative diseases PD. One promising metal modulator is ATH434 (formerly PBT434). ATH434 is an 8-hydroxyquinazolinone with a moderate binding affinity for the physiological transition metals iron, copper, and zinc [22]. ATH434 is able to form complexes with the labile pool of metals arising from loss of metal homeostasis in disease. This moderate binding affinity is superior to high-affinity chelators, as it does not disrupt physiological metal homeostasis, but inhibits redox activity initiated by unregulated copper and iron [22]. Similarly, 5-Chloro-7-iodo-quinolin-8-ol (clioquinol) has been shown to reduce iron levels, prevent neurodegeneration, and rescue motor impairment in aged tau^−/−^ mice [23]. Given the clear involvement of metals in nigral pathological processes in PD, we hypothesize that metal dyshomeostasis contributes to olfactory dysfunction in PD. The present study resolves to test this hypothesis by examining the status of disease-related transitional metals in tau^−/−^ mice and testing the effect of ATH434 on olfactory and motor phenotypes in young and aged mice.

## Methods

### Animals

Mice were group-housed in standard transparent individually ventilated cages (IVC; 29.5 × 16 × 13 cm) on sawdust under a 12 h light/dark cycle (lights on at 0700 h). All testing was performed during the light phase of the circadian cycle. Rodent chow and water were available *ad libitum*. Sv129B/6 tau^−/−^ mice (initially described by Dawson et al. [24]) and wild-type controls (WT) were bred in-house. All mice were genotyped using a standardized polymerase chain reaction assay for tail DNA (Transnetyx Inc., USA). Animal information including age and sex are reported in Supplementary Table 1. All studies were conducted in a blinded fashion. All methods conformed to the Australian National Health and Medical Research Council published code of practice for animal research and all experimentation was approved by The Florey Animal Ethics Committee (AEC number: 15–092). All efforts were made to ensure comfort and minimize the suffering of animals throughout.

### ATH434 Treatment

ATH434 (formerly PBT434) was kindly provided by Alterity Therapeutics (formerly Prana Biotechnology). ATH434 was sonicated in standard suspension vehicle (SSV) (0.9% w/v sodium chloride, 0.5% w/v sodium carboxymethylcellulose, 0.5% v/v benzyl alcohol, 0.4% v/v Tween 80) 30 min before oral gavage dosing.

5.5-month-old WT and tau^−/−^ and 13.5-month-old WT and tau^−/−^ animals underwent 6 weeks of daily oral gavage treatment of ATH434 (30 mg/kg/day) or SSV. For the subsequent 7 days, animals underwent one behavioral task per day, receiving treatment immediately after completing the behavioral task each day. Behavioral tasks were performed in the order: odor detection test (ODT), Rota Rod (2 days), and pole test (2 days). The following day animals were dosed 30 min before euthanasia.

### Animal Behavior

#### Odor Detection Test

The ODT was adapted from a previously described protocol [25]. Mice were habituated to vehicle canisters for 3 days before testing. The test day (day 4) comprised of three 5-min trials (1-h inter-trial interval (ITI)) performed in the home cage in which the mice were exposed to two visually identical canisters; one vehicle (400 µL, MilliQ water + 0.1% Tween20) and one novel odor canister of either 0 (vehicle) or 1:10^4^ dilutions (400 µL, MilliQ water + 0.1% Tween20 + orange essential oil (In Essence, Aus)). Animals were filmed and videos were manually scored (the scorer was blinded to experimental conditions) and the percentage of investigation time was calculated based on: (time spent investigating novel odor canister ÷ time spent investigating both canisters) × 100. Normal mice will spend more time investigating a novel odor; as such, this test, assuming no cognitive or motor impairments, determines the concentration at which mice can detect a novel odor by comparing the time spent investigating the two canisters.

#### Rotarod

Motor coordination and ataxia were assessed in mice via the Rota Rod test [26]. Mice were trained for three sessions on the rotarod (Panlab, Spain) 24 h before testing. Session 1 and session 2 were at fixed speeds (4 rpm for 2 min each), and session 3 was accelerating (4–40 rpm for 2 min). During training, if the mouse fell off the rotarod it was placed back on until 2 min had elapsed. On the test day, the Rota Rod was set to accelerating mode (4–40 rpm) over a 5 min trial. Mice were allowed three attempts and the average time of latency to fall was recorded.

#### Pole Test

Motor coordination was assessed using the pole test [27]. Mice were loosely scruffed and placed vertically (nose up) on a pole (50 cm) that was wrapped in a self-adhesive bandage (NexCare, Aus). A small hard rubber ball was placed on top of the pole to prevent animals from climbing and sitting on the top. Two lines were drawn on the pole to delineate a 40-cm segment of the pole. One day before testing, animals were habituated to the pole and successfully completed five trials. On the day of testing, animals were allowed three consecutive attempts which were recorded. Time to turn (animal to complete a 180° rotation) and time to descend (time from the beginning of turn to nose crossing the distal line) was recorded.

### Tissue Preparation

After behavioral analysis, animals were terminally anaesthetized using 100 mg/kg intraperitoneal pentobarbitone (Virbac, Aus) injection and transcardially perfused with Dulbecco’s phosphate-buffered saline (D-PBS) (Gibco, Aus) containing 55.6 mg/L heparin (Sigma Aldrich, Aus) with approximately 3 times their blood volume (7% of body weight).

Brains were collected and the left hemisphere was micro-dissected to collect the OB and SNpc before being frozen on dry ice and stored at −80 °C. Frozen sections were homogenized using a probe sonicator (10 s) in PBS (Sigma-Aldrich, USA) with protease and phosphatase inhibitors (Complete Mini Protease Inhibitor Cocktail & PhosSTOP Phosphatase Inhibitor Cocktail, Roche Diagnostics, USA). Homogenates were then centrifuged at 10,000 *g* for 20 min at 4 °C. The clarified supernatant was collected (cell lysate) and total protein concentrations were determined using the bicinchoninic acid assay (Pierce, USA) according to the manufacturer’s directions.

The right hemisphere was placed in 50 mL 4% paraformaldehyde for post-fixation. After 24 h brains were transferred to 30% white sugar (CSR, Aus) solution (in D-PBS) and stored at 4 °C overnight. Brains were then transferred to a fresh 30% white sugar solution and stored at 4 °C for one week before being snap-frozen with isopentane and stored at –80 °C.

### Inductively Coupled Plasma Mass Spectrometry

Inductively coupled plasma mass spectrometry (ICP-MS) was performed as previously reported [23]. Briefly, samples were lyophilized and nitric acid (65% Suprapur, Merck, USA) was added for 6-h digestion at room temperature. Samples were heated at 90 °C for 20 min followed by the addition of hydrogen peroxide (30% Aristart, BDH; UAE). Samples were left at room temperature for 30 min before heating again for a further 15 min at 70 °C. The average reduced volume was determined, and the samples were further diluted with 1% HNO_3_ diluent. Measurements were made using an Agilent 7700 series ICP-MS instrument under routine multi-element operating conditions using a Helium Reaction Gas Cell. The instrument was calibrated using 0, 5, 10, 50, 100, and 500 ppb of certified multi-element ICP-MS standard calibration solutions (ICP-MS-CAL2-1, ICP-MS-CAL-3, and ICP-MS-CAL-4, Accustandard; USA) for a range of elements. Certified internal standard solutions containing 20 ppb of Yttrium (Y89) as an internal control (ICP-MS-IS-MIX1-1, Accustandard, USA) were used.

### SDS-PAGE and Immunoblot Analysis

Homogenized samples were thawed on ice and mixed with 4 X sample buffer (0.25 M Tris, 8% SDS, 20% glycerol, 0.4% bromophenol blue) containing 10% 1 M dithiothreitol (DTT), boiled for 5 min, and centrifuged at 10,000 × *g* for 5 min. Protein was electrophoresed at 270 V for 25 min on 4–20% polyacrylamide gels (BioRad, Aus). Gel transfer was performed using the iBlot 2 (Invitrogen, USA) system.

#### Fluorescence Detection

Membranes (0.45 µm nitrocellulose, BioRad, USA) were blocked in Blocker™ FL Fluorescent Blocking Buffer (ThermoFischer, Aus) for 1 h at RT. All antibodies (α-syn (1:5000), BD biosciences; β-actin (1:10,000), CST; synaptophysin (1:5000), CST) were diluted in TBS-T. Membranes were washed in TBS-T for 21 min (3 × 7 min) before and after incubation with fluorescent secondary antibodies (Licor, Aus). Proteins were visualized with the Licor Odyssey fc system (Licor, Aus) and analyzed via signal intensities (ImageStudio 5.2, Licor, Aus). Samples normalized to β-actin, except for 4-HNE which was normalized to total protein as determined by Revert Total Protein Staining (Licor, Aus) according to manufacturer’s direction.

### Stereology and TH Immunohistochemistry

The SNpc was sectioned in a 1 in 3 series at 30 μm with a cryostat (Leica, USA).

#### Stereology

Sections were stained with 1% neutral red (Nissl). The number of neurons in the SNpc was estimated using a fractionator sampling design as described previously [28]. Briefly, counts were made at regular intervals (*X* = 140 µm, *Y* = 140 µm). Systematic samples of the area occupied by the nuclei were made from a random starting point. An unbiased counting frame of known area (45 μm × 45 μm) was superimposed on the image of the tissue sections using stereology software (MBF, Stereo Investigator) utilizing a 63 × objective lens (Leica, N.A.1.36). Experimenters were blinded to the treatments of each of the groups. The entire SNpc nucleus was sampled to obtain an estimate of neuronal number.

#### TH Immunohistochemistry

Sections from the same animals that underwent Nissl staining were then processed for TH immunohistochemistry. Frozen sections were defrosted for 3 h at room temperature. Sections were blocked in 3% normal goat serum (NGS) with 0.3% Triton-X for 30 min. Sections were then placed in primary antibody solution (1:1000; Millipore) comprising of 1% NGS and 0.15% Triton X overnight at 4 °C. Slides were washed in PBS and incubated in polyclonal goat anti-rabbit HRP-conjugated secondary antibody solution (1:200; Dako) overnight at 4 °C. Slides are then washed in PBS and incubated in diamino-benzidine (DAB) solution comprising of 1% DAB, 1% cobalt chloride and 1% nickel sulphate in 0.2 M phosphate buffer for 20 min at room temperature. H_2_O_2_ (3%) was added to DAB solution for 3 min before additional washes in PBS. Sections were then counterstained in Nissl substance (1% neutral red) for 2 min and dehydrated through increasing concentrations of ethanol (3 min of each; 0%, 50%, 70%, 90%, 100%), cleared in xylene (Sigma-Aldrich, Aus) and cover slipped. Slides were imaged and counted as per the stereology protocol, where cells that were both Nissl^+^ and TH^+^ were counted as dopaminergic neurons.

### Statistical Analysis

For all statistical analyses, the software package GraphPad Prism (version 6.05 for Windows) was used. Detail including statistical test, replicate number, experimental repeats, and significance are reported in figures. ANOVA *P* values reported are from post hoc comparisons generated only when ANOVA terms were significant. One-sample *t*-tests were also performed for ODT results to determine if the animals could detect odors at specified concentrations (independent of genotype), as evidenced by spending significantly more than 50% of the investigation time with an odorous canister. An investigation time of 50% represents chance, indicating an animal cannot differentiate between the canisters, i.e. cannot detect an odor. For all analyses, *P* < 0.05 was considered statistically significant.

## Results

### ATH434 Prevents Hyposmia in 7-Month Old Tau^−/−^ Mice

**Fig. 1:**
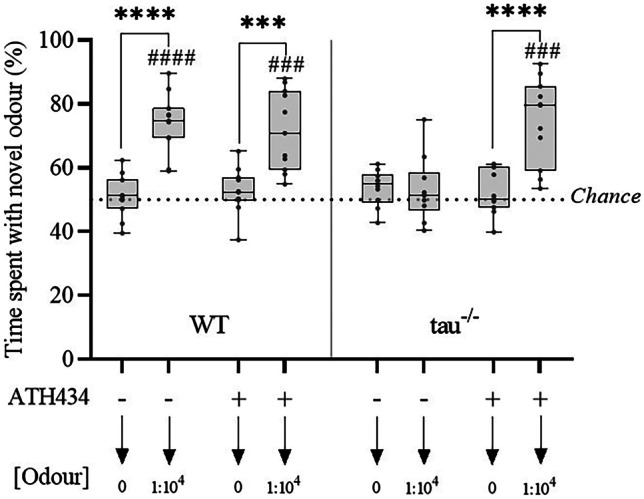
ATH434 prevents hyposmia in 7-month-old tau^−/−^ mice. An odor detection test was performed on 7-month-old WT and tau^−/−^ mice following 6-week treatment with either SSV or ATH434. WT (SSV) *N* = 11; WT (ATH434) *N* = 11; tau^−/−^ (SSV) *N* = 10; tau^−/−^ (ATH434) *N* = 11. Analysis was performed by three-way ANOVA with Sidak post hoc comparisons (denoted by asterisks). ***P < 0.001, ****P < 0.0001. Secondary analysis was performed by a one-sample *t*-test with a hypothetical mean of 50% (denoted by octothorpes). ^###^*P* < 0.001, ^####^*P* < 0.0001

Following 6 weeks of treatment with either ATH434 or SSV, 7-month-old tau^−/−^ and WT mice underwent an ODT which demonstrated a significant olfactory impairment in untreated tau^−/−^, which was rescued by treatment with ATH434 (Fig. 1). A three-way ANOVA revealed a significant effect for all interactions (Supplementary Table 2). Importantly, this phenotype emerged in the *absence of overt motor dysfunction* in the mice (as measured by the rotarod and pole test), making it a pre-motor impairment (Supplementary Fig. 1).

Subsequent multiple comparison analysis demonstrated a significant increase in investigation time of the odorous canister in WT mice treated with vehicle and ATH434 (*P* < 0.0001 and *P* = 0.003, respectively) and ATH434 treated tau^−/−^ mice (*P* < 0.0001). This indicated that the WT could detect the odor and treatment normalized that ability that was lost in untreated tau^−/−^ mice who showed no preference for investigating a novel odor, indicating that their olfactory abilities were impaired. In contrast, ATH434 treated tau^−/−^ mice had a significantly increased relative investigation time to the odorous canister compared to the control canister (*P* < 0.0001), indicating that treatment had rescued the olfactory performance of the tau^−/−^ mice. A one-sample *t*-test was performed on all trials to determine the significance of odor investigation compared to a hypothetical mean of 50% (chance). During the odorous trial, WT (SSV and ATH434) treated mice and ATH434 treated tau^−/−^ mice performed significantly better than chance (*P* < 0.0001, *P* = 0.002, *P* = 0.002, respectively).

### ATH434 Alters Metal and Protein Levels in the Tau^−/−^ Olfactory Bulb

**Fig. 2:**
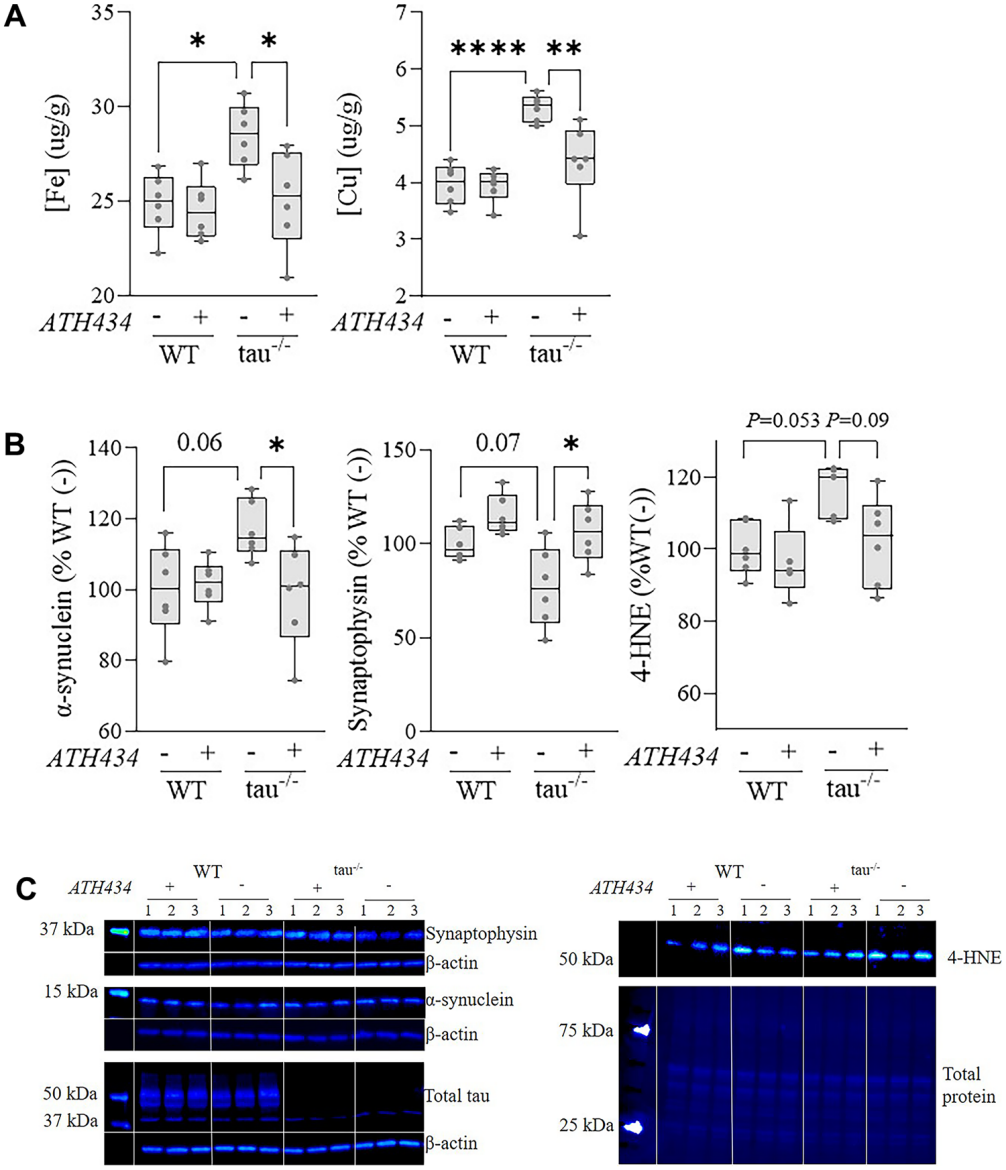
ATH434 normalizes bulbar metal and protein changes in 7-month-old tau^−/−^ mice. **A** Iron (Fe) and copper (Cu) ICP-MS analysis was performed on 7-month-old WT and tau^−/−^ olfactory bulbs following 6-week treatment with either SSV or ATH434. **B** Quantification of western blot densitometry presented as % of α-syn or synaptophysin relative to untreated WT control. OB lysate for western blots was normalized to β-actin from three independent replicates. **C** Representative western blots of olfactory bulb cell lysate from 7-month-old WT and tau.^−/−^ immunoblotted for α-syn, synaptophysin, total tau, 4-HNE, and β-actin/total protein. *N* = 6/group. Analyses were performed by two-way ANOVA with Tukey post hoc comparisons. *P < 0.05, **P < 0.01, ***P < 0.001, ****P < 0.0001

The levels of bulbar metals and synaptic markers were compared between 7-month-old SSV and ATH434 treated WT and tau^−/−^ mice. A two-way ANOVA with Tukey post hoc test for multiple comparisons was performed for all analyses, factors, and interactions reported in Supplementary Table 3. Untreated tau^−/−^ mice had significantly increased levels of iron (*P* = 0.02), which were reduced by ATH434 treatment (*P* = 0.03). An analysis of copper showed that untreated tau^−/−^ mice had significantly increased levels (*P* < 0.0001) that were reduced by ATH434 treatment (*P* = 0.002) (Fig. 2a). ATH434 neither increased nor reduced metal concentrations in WT mice.

α-syn and synaptophysin levels were analyzed in the OB by SDS-PAGE western blot. A two-way ANOVA revealed an increase in monomeric α-syn in the untreated tau^−/−^ mice, although this did not reach significance (*P* = 0.06), and ATH434 treatment significantly reduced α-syn level in the tau^−/−^ mice (*P* = 0.04). There was also a decrease in synaptophysin, although this did not reach significance (*P* = 0.07), but ATH434 treatment significantly increased bulbar synaptophysin in tau^−/−^ mice (*P* = 0.01) (Fig. 2b, c) ATH434 neither increased nor reduced α-syn or synaptophysin levels in WT mice. Tau ablation was confirmed by immunoblot (Fig. 2c). Finally, 4-hydroxynonenal (4-HNE) was measured and a two-way ANOVA revealed an increase in tau^−/−^ OB (*P* = 0.06). ATH434 decreased 4-HNE levels in the tau^−/−^ OB but this did not reach significance (*P* = 0.09). Full immunoblots are available (Supplementary Fig. 2).

### ATH434 Rescues Motor Impairments in 15-Month Old Tau^−/−^ Mice

**Fig. 3:**
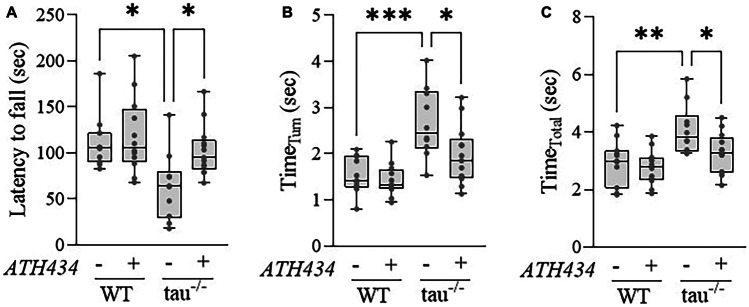
ATH434 rescues the motor impairment in 15-month-old tau^−/−^ mice. **A** Latency to fall on the rotarod, **B** time to turn on the pole test, **C** time to complete the pole test performed on 15-month-old WT and tau^−/−^ mice following 6-week treatment with either SSV or ATH434. WT (SSV) *N* = 11; WT (ATH434) *N* = 12; tau^−/−^ (SSV) *N* = 10; tau.^−/−^ (ATH434) *N* = 13. Analysis was performed by two-way ANOVA with Tukey’s post hoc test for multiple comparisons. *P < 0.05, **P < 0.01, ***P < 0.001

Following the benefit of ATH434 treatment in rescuing the pre-motor hyposmia in 7-month-old tau^−/−^ mice, we sought to examine the effect of treatment on the previously reported motor impairment in aged (15-month old) mice [13]. After 6 weeks of treatment with either ATH434 or SSV, 15-month-old tau^−/−^ and WT mice undertook rotarod and pole tests, which demonstrated a significant motor impairment in untreated tau^−/−^ that were rescued by ATH434 (Fig. 3).

On the rotarod, untreated tau^−/−^ had a significantly reduced latency to fall (*P* = 0.01) which was rescued by ATH434 (*P* = 0.04) (Fig. 3a). The pole test was measured by the time to turn and the time to complete the task (total time). Untreated tau^−/−^ mice had a significantly increased time to turn (*P* = 0.0002) which was rescued by ATH434 treatment (*P* = 0.02) (Fig. 3b). Untreated tau^−/−^ mice had a significantly increased total task time (*P* = 0.004) which was rescued by ATH434 treatment (*P* = 0.04) (Fig. 3c). ATh434 had no effect on motor indices in WT mice.

### ATH434 Reduces Iron and Preserves Nigral Neurons in 15-Month Old Tau^−/−^ Mice

**Fig. 4:**
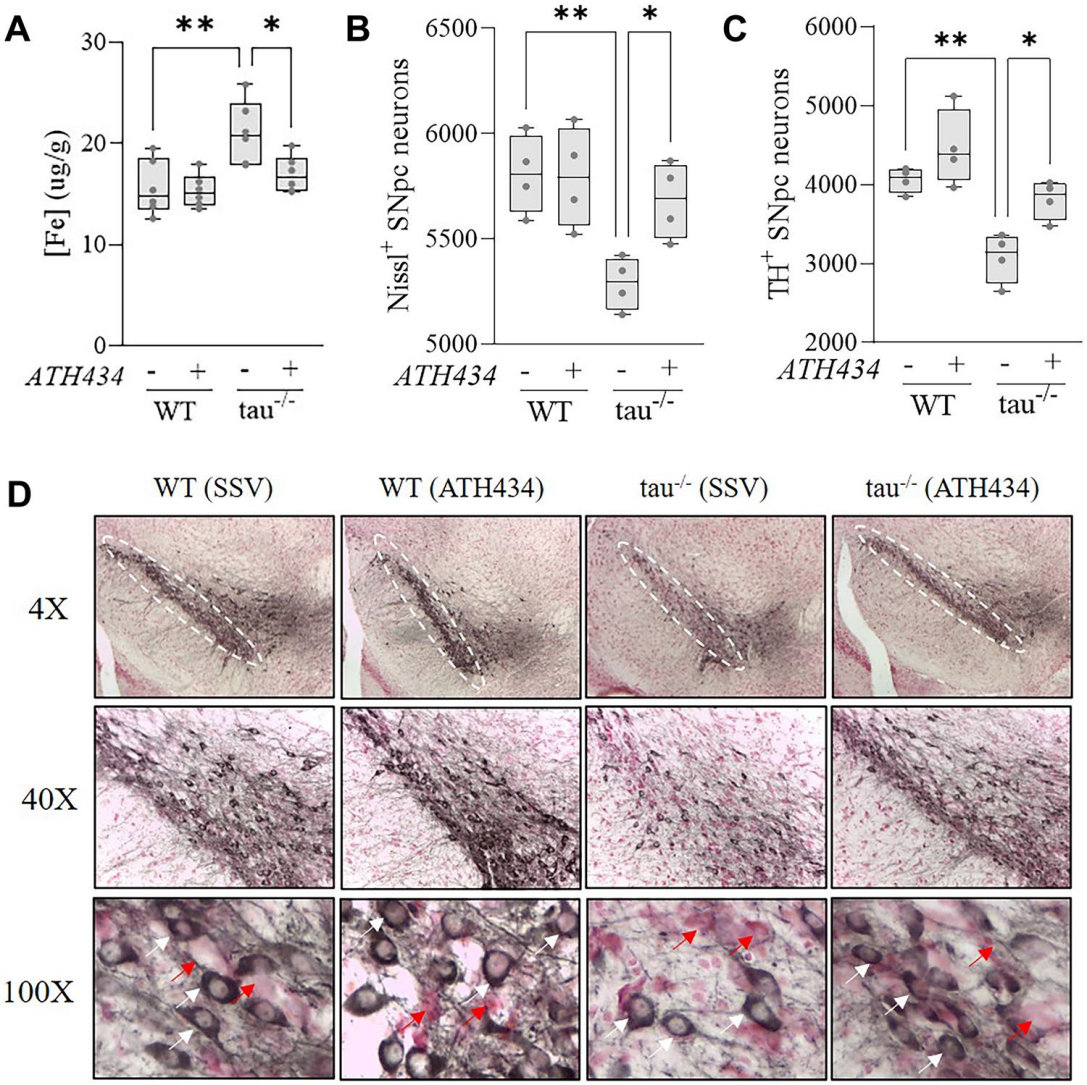
ATH434 reduces iron accumulation and protects against neurodegeneration in the SNpc of 15-month-old tau^−/−^ mice. **A** Fe ICP-MS analysis (*N* = 6/group) and **B** SNpc stereology neuron counts (*N* = 4/group) and **C** TH immunohistochemistry counts (*N* = 4/group) were performed on 15-month-old WT and tau^−/−^ SNpc following 6-week treatment with either SSV or ATH434. **D** Representative sections of SNpc stained for TH and Nissl at × 4, × 40, and × 100 magnification. Analysis was performed by two-way ANOVA with Tukey’s post hoc test for multiple comparisons. White ovals signify SNpc (region of interest). Red arrows identify TH^−^/Nissl^+^ cells and white arrows identify TH^+^/Nissl.^+^ cells. *P < 0.05, **P < 0.01, ***P < 0.001

An increase in iron and loss of nigral neurons have been previously reported to underlie the motor deficits in the tau^−/−^ mice [14]. As such, we examined the SNpc of WT and tau^−/−^ mice for iron concentration as well as total neuron and dopaminergic neuron counts. There was a significant increase in Fe in the SNpc of untreated tau^−/−^ mice (*P* = 0.003) which was normalized by ATH434 treatment (*P* = 0.03) (Fig. 4a). Untreated tau^−/−^ had a significant loss in SNpc neurons (*P* = 0.005) which were protected by ATH434 treatment (*P* = 0.048) (Fig. 4b). Untreated tau^−/−^ mice had a significant loss in dopaminergic neurons (*P* = 0.006) which were protected by ATH434 treatment (*P* = 0.04) (Fig. 4c). Representative Nissl^+^/TH^+^ stained SNpc sections are presented in Fig. 4d. Neither the genotype nor ATH434 treatment altered α-syn or copper levels in the SNpc of aged tau^−/−^ mice (data not shown).

## Discussion

The tau^−/−^ mice have been proposed as an age-dependent model of parkinsonism as they develop a motor impairment that is associated with degeneration of nigrostriatal neurons and accumulation of nigral iron [14], key features of PD. Tau plays an integral role in microtubule stability and axonal transport, and tau^−/−^ mice are reported to accumulate metals as a consequence of failure to adequately traffic key metal efflux proteins [14]. As we have previously reported [13], tau^−/−^ animals have an olfactory impairment at 7 months of age, before the onset of the motor impairment at 15 months, modeling a key prodromal feature of PD. These findings have been reproduced in the second cohort of tau^−/−^ mice in this study, confirming a significant olfactory deficit in the absence of overt motor impairment at 7-months.

An increase in Fe in the SNpc is well-established in PD [16, 17, 29–31], and metal modulation with iodochlorhydroxyquin (clioquinol) has shown therapeutic benefit by reducing Fe, preventing neurodegeneration, and rescuing the motor impairment in aged tau^−/−^ mice [23]. To investigate the contribution of elevated metals in olfactory dysfunction and the effects of ATH434 in preventing hyposmia, 5.5-month-old tau^−/−^ mice and WT controls were treated for 6 weeks. Our previous characterization of this mouse model at 4 months of age demonstrated a reduction in odour detection was observable in some mice within the cohort, suggesting that the impairments in olfactory performance had begun, however a robust impairment across all animals within a cohort was not observed until 7 months of age. As such, we began treatment in the 6 weeks prior to the age of a robust presentation of hyposmia. Following treatment, tau^−/−^ mice had intact olfaction compared to their untreated counterparts which was aligned with the normalization of iron and copper levels in the OB. These data suggest that metal accumulation may have functional outcomes in olfactory processing which can be relieved by re-establishing metal homeostasis. The precise role of metals in olfactory function, or dysfunction, is not well understood. A small study has associated the accumulation of bulbar iron in humans with hyposmia [32]. Interestingly, while iron is well characterized in PD, we found a significant increase in copper in the OB. Copper is found in relatively high concentrations within Lewy bodies and is effective at causing fibrillation and ‘seeding’ of protein aggregates [33]. Additionally, it is a key cofactor in cellular antioxidants known to be altered in PD [34–39] and copper accumulation has been reported to affect the olfactory capacity of various fish species [40–42].

The normalization of metal levels following ATH434 treatment coincided with normalization of α-syn and synaptophysin protein levels. Iron-mediated reductions in synaptophysin have been previously reported, and reduction of iron levels resulted in restoration of synaptophysin levels and functional readout (cognitive) [43]. Additionally, metal chelation has been shown to increase the generation of synaptophysin-containing processes in a toxin model of parkinsonism [44]. These data suggest that the accumulation of bulbar metals may be detrimental to synaptophysin levels and that normalizing metal homeostasis restores synaptic viability contributing to improved olfaction. The OB is a complex structure that consists of projection neurons, interneurons, and centrifugal input from cortical structures. Future studies may work to uncover the potential regionality of metal accumulation and synaptophysin reduction in the tau^−/−^ mice. In human PD OB tissue, there is a significant increase in iron in the mitral cell and external plexiform layers, and copper is increased in the external plexiform, mitral cell, and glomerular layers [19]. Identifying the neuronal populations susceptible to metal-related changes in this model may provide insight into precision interventions.

Given the restoration of hyposmia with ATH434, we then tested aged tau^−/−^ animals mice that express motor impairments. 13.5-month-old aged tau^−/−^ mice had an overt motor impairment as measured by the rotarod and pole test, and these motor phenotypes were functionally rescued by 6 weeks’ treatment with ATH434. The restoration of motor impairment was aligned with a reduction in iron and protection against SNpc neurodegeneration. These changes underlie a functional rescue of the midbrain phenotype in tau^−/−^ mice and align well with previous reports of the effects of ATH434 [45]. There were no nigral changes in copper, suggesting copper may be regionally altered in disease. Additionally, there was no increase in nigral α-syn which aligns with our previous findings that α-syn is accumulated in the caudate putamen but not the SNpc of aged tau^−/−^ mice [13]. These data support a growing body of literature that emphasizes the neuroprotective effects of iron modulators in PD such as apomorphine, clioquinol, deferoxamine, M30, and VK-28 [46–51]. Furthermore, ATH434 has been shown to inhibit iron-mediated redox activity and rescue motor impairments in the 6-OHDA, MPTP, and an α-syn genetic mouse model of PD previously [28], and this work indicates efficacy in a prodromal PD model.

Nigral metal dyshomeostasis is involved in the pathogenesis of PD, as it leads to increased oxidative stress. As a consequence, metal modulation has become of interest as a therapeutic strategy, with more than 250 compounds with metal-binding affinity being tested in models of PD between 2014–2019 [52]. Aged tau^−/−^ mice have increased nigral iron and 4-HNE (a marker of lipid peroxidation and indicator of oxidative stress [53]; Supplementary Fig. 3) which are restored by ATH434. Importantly, this study demonstrates that metal homeostasis is also perturbed in the OB of young, hyposmic tau^−/−^ animals who have a concurrent increase in 4-HNE. As far as we are aware, the data reported here are the first demonstration of pharmacological restoration of pre-motor parkinsonian hyposmia in vivo. As such, these findings support our hypothesis that disruption of metal homeostasis plays a role in prodromal olfactory impairments. Olfactory testing may be a useful readout to be incorporated into clinical trials with metal modulating agents, and ATH434 may have therapeutic potential in prodromal PD.

## Supplementary Information

Below is the link to the electronic supplementary material.

Supplementary file1 (PDF 1225 KB)

Supplementary file2 (PDF 1225 KB)

Supplementary file3 (PDF 1225 KB)

Supplementary file4 (PDF 1225 KB)

Supplementary file5 (PDF 1225 KB)

Supplementary file6 (PDF 1225 KB)

Supplementary file7 (PDF 1225 KB)

Supplementary file8 (DOCX 2405 KB)
