# Supplementary material for: ATH434 Rescues Pre-motor Hyposmia in a Mouse Model of Parkinsonism
Source: Neurotherapeutics. 2022 Sep 29;19(6):1966–75. doi: 10.1007/s13311-022-01300-0 (PMC9723006; doi:10.1007/s13311-022-01300-0)
Supplement: Supplementary file 8 — Supplementary file8 (DOCX 2405 KB) [file 13311_2022_1300_MOESM8_ESM.docx]

**SUPPLEMENTARY DATA**

**Supplementary Table 1. Animal information**

|  |  |  | **Behavioural tests** | | **Biological tests** | |
| --- | --- | --- | --- | --- | --- | --- |
| **Age** | **Group** | **Drug** | ***N* (female)** | ***N* (male)** | ***N* (female)** | ***N* (male)** |
| 5.5-7 mo | WT | SSV | 6 | 5 | 3 | 3 |
|  |  | ATH434 | 5 | 6 | 3 | 3 |
|  | Tau^-/-^ | SSV | 5 | 5 | 3 | 3 |
|  |  | ATH434 | 6 | 5 | 3 | 3 |
| 13.5-15 mo | WT | SSV | 5 | 6 | 3 | 3 |
|  |  | ATH434 | 6 | 6 | 3 | 3 |
|  | Tau^-/-^ | SSV | 5 | 5 | 3 | 3 |
|  |  | ATH434 | 6 | 7 | 3 | 3 |

**Supplementary Table 2.** Three-way ANOVA results for ODT

| **Source** *(Dependent variable: Genotype)* | **F (DFn, DFd)** | ***P-*value** |
| --- | --- | --- |
| Treatment | F (1, 40) = 4.687 | **0.036** |
| Genotype | F (1, 38) = 5.414 | **0.025** |
| Odor concentration | F (1, 40) = 47.33 | **< 0.0001** |
| Treatment x genotype | F (1, 38) = 9.515 | **0.0038** |
| Treatment x odor concentration | F (1, 40) = 4.429 | **0.042** |
| Genotype x odor concentration | F (1, 38) = 9.167 | **0.0044** |
| Treatment x Genotype x odor concentration | F (1, 38) = 15.73 | **0.0003** |

**Supplementary Table 3.** Two-way ANOVA results

| **Analysis** | **Source** | **F (DFn, DFd)** | ***P*-value** |
| --- | --- | --- | --- |
| [Fe] (OB) | Treatment | F (1, 20) = 5.711 | **0.0268** |
|  | Genotype | F (1, 20) = 7.135 | **0.0147** |
|  | Treatment x genotype | F (1, 20) = 3.844 | 0.0640 |
| [Cu] (OB) | Treatment | F (1, 20) = 10.24 | **0.0045** |
|  | Genotype | F (1, 20) = 45.41 | **<0.0001** |
|  | Treatment x genotype | F (1, 20) = 8.892 | **0.0074** |
| α-syn (OB) | Treatment | F (1, 20) = 3.389 | 0.0805 |
|  | Genotype | F (1, 20) = 2.374 | 0.1390 |
|  | Treatment x genotype | F (1, 20) = 4.840 | **0.0397** |
| Synaptophysin (OB) | Treatment | F (1, 20) = 13.44 | **0.0015** |
|  | Genotype | F (1, 20) = 6.696 | **0.0175** |
|  | Treatment x genotype | F (1, 20) = 1.335 | 0.2615 |
| 4-HNE (OB) | Treatment | F (1, 20) = 4.540 | **0.0472** |
|  | Genotype | F (1, 20) = 7.073 | **0.0160** |
|  | Treatment x genotype | F (1, 20) = 1.689 | 0.2101 |
| Rota Rod | Treatment | F (1, 42) = 6.562 | **0.0141** |
|  | Genotype | F (1, 42) = 11.84 | **0.0013** |
|  | Treatment x genotype | F (1, 42) = 1.822 | 0.1843 |
| Pole Test (turn) | Treatment | F (1, 42) = 6.803 | **0.0125** |
|  | Genotype | F (1, 42) = 26.86 | **<0.0001** |
|  | Treatment x genotype | F (1, 42) = 2.940 | 0.0938 |
| Pole Test (total) | Treatment | F (1, 42) = 6.007 | **0.0185** |
|  | Genotype | F (1, 42) = 15.59 | **0.0003** |
|  | Treatment x genotype | F (1, 42) = 2.029 | 0.1617 |
| [Fe] (Snpc) | Treatment | F (1, 20) = 5.655 | **0.0275** |
|  | Genotype | F (1, 20) = 14.37 | **0.0011** |
|  | Treatment x genotype | F (1, 20) = 3.617 | 0.0717 |
| Neuronal Counts | Treatment | F (1, 12) = 4.258 | 0.0614 |
|  | Genotype | F (1, 12) = 14.87 | **0.0023** |
|  | Treatment x genotype | F (1, 12) = 4.705 | 0.0509 |
| TH^+^ Counts | Treatment | F (1, 12) = 12.45 | 0.004 |
|  | Genotype | F (1, 12) = 25.78 | 0.0003 |
|  | Treatment x genotype | F (1, 12) = 1.035 | 0.3291 |


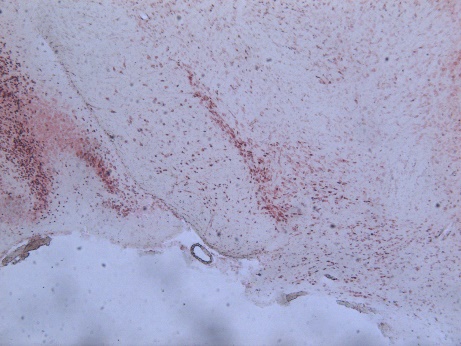

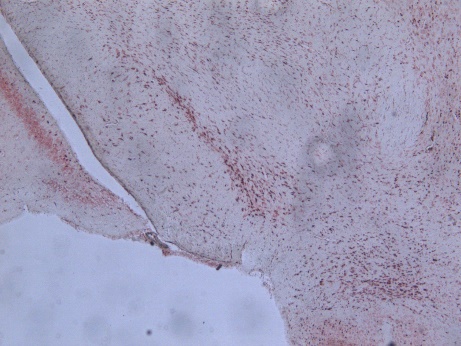

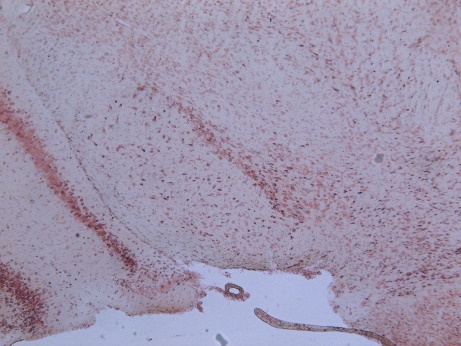

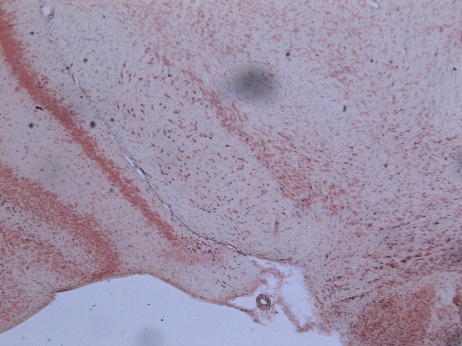


a

b

c

d

e

WT SSV

tau^-/-^ SSV

tau^-/-^ ATH434

WT ATH434

**Supplementary Fig. 1** There is no motor impairment or nigral alterations in the 7-month-old tau^-/-^ mice. A) Latency to fall on the rotarod, B) time to turn and time to complete the pole test; WT SSV (*N*=11), WT ATH434 (*N*=11), tau^-/-^ SSV (*N*=10), tau^-/-^ ATH434 (*N*=11). C) Fe ICP-MS analysis (*N*=6/group), D) SNpc stereology neuron counts (*N*=4/group) and E) representative sections of SNpc stained for neutral red. Analysis was performed by two-way ANOVA with Tukey’s posthoc test for multiple comparisons. White ovals signify SNpc (region of interest)

**
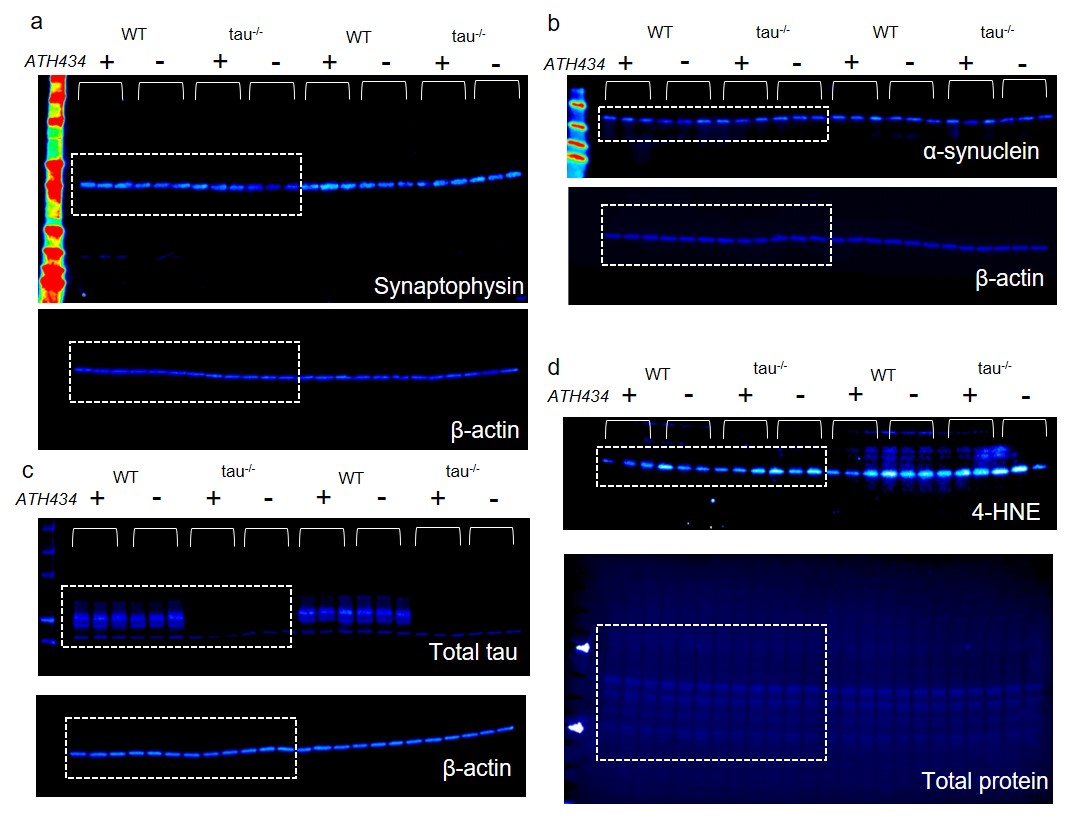
**

**Supplementary Fig. 2** Full immunoblots from 7-month-old WT and tau^-/-^ ATH434 (+) and SSV (-) treated olfactory bulb tissue. A) Synaptophysin (anti-rabbit) and β-actin (anti-mouse), B) α-synuclein (anti-mouse) and β-actin (anti-mouse), C) total tau (anti-rabbit) and β-actin (anti-mouse), D) 4-HNE (anti-rabbit) and total protein *N*=6/group, white perforated boxes indicate representative bands presented in the manuscript

Supplementary Fig. 3 4-HNE immunoblot from 15-month-old WT and tau-/- ATH434 (+) and SSV (-) treated SNpc tissue. A) Full 4-HNE (anti-rabbit) and total protein blots, B) quantification of immunoblot by one-way ANOVA. N=6/group, * *P*<0.05, *** *P*<0.001


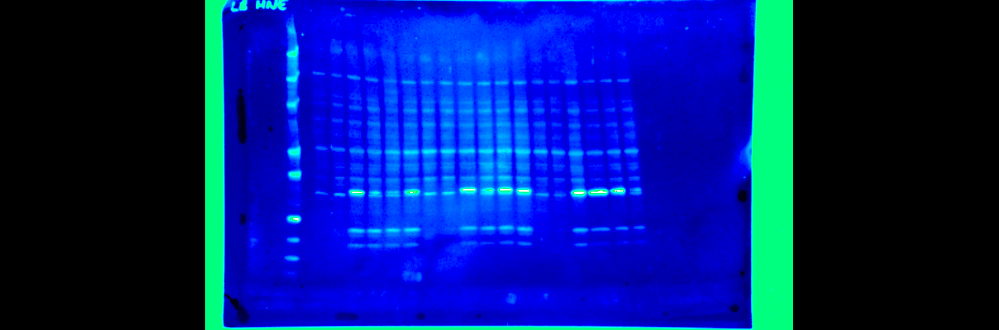

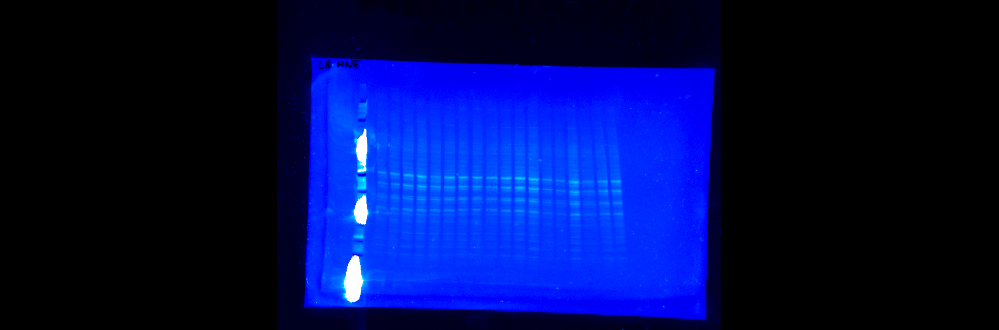


WT(-)

WT(-)

WT(-)

KO(-)

KO(-)

KO(-)

KO(+)

KO(+)

KO(+)

4-HNE

Total Protein

a

b
